# Factors Associated With Skilled Birth Attendance Among Internally Displaced Women in Mogadishu, Somalia: A Community‐Based Cross‐Sectional Study

**DOI:** 10.1002/hsr2.73379

**Published:** 2026-10-13

**Authors:** Maryama Ahmed Ali, Yusuf Hared Abdi, Abdisalan Abdikarin Mohamed, Yaxye Abuukar Hassan, Abdiqani Mohamed jama, Sadio Abdulahi Osman, Sharmake Gaiye Bashir, Yakub Burhan Abdullahi, Naima Ibrahim Ahmed, Ilyas Abdullahi khalif, Mohamed Sharif AbdI, Ahmed Abdinasir Abdulle, Nuradin Abdullahi Sheikh Rashid

**Affiliations:** ^1^ Faculty of Health Sciences and Tropical Medicine Somali National University Mogadishu Somalia; ^2^ Center for Health Research and Innovation Somali National University Mogadishu Somalia; ^3^ Benadir Research Consultancy and Evaluation Center (BRCE) Mogadishu Somalia; ^4^ Department of Public Health, Faculty of Medicine and Health Sciences Hormuud University Mogadishu Somalia; ^5^ Faculty of Health Science Salaam University Mogadishu Somalia

## Abstract

**Background and Aims:**

Internally displaced women in Somalia face major barriers to skilled maternal care. This study estimated the prevalence of skilled birth attendance (SBA) and identified factors associated with SBA among women living in internally displaced persons (IDP) camps in Mogadishu.

**Methods:**

A community‐based cross‐sectional study was conducted among women aged 15–49 years in IDP camps in Deynile, Kahda, and Garasbaaley districts, Mogadishu, in February 2026, using multistage sampling and interviewer‐administered questionnaires. The outcome was SBA at the most recent birth. Multivariable logistic regression estimated adjusted odds ratios (AORs) with 95% confidence intervals (CIs); tests were two‐sided (α = 0.05).

**Results:**

Of 384 women interviewed, 382 were eligible. SBA was reported by 68 of 381 women (17.8%; 95% CI: 14.3–22.0); 295 (77.4%) were assisted by traditional birth attendants and 255 of 382 (66.8%) delivered at home. Only 42 of 370 women (11.4%) received any antenatal care (ANC). In the adjusted model (n = 366; 67 events), SBA was associated with ANC attendance (AOR 3.17; 95% CI: 1.27–7.94) and Quranic education compared with no education (AOR 3.13; 95% CI: 1.61–6.10). Estimates for primary education (6 women; AOR 6.13; 95% CI: 1.05–35.91) and public transport (31 women; AOR 0.14; 95% CI: 0.02–0.77) were imprecise. The model discriminated well (AUC 0.79) but had few events per parameter (4.5).

**Conclusion:**

SBA was very low among displaced women in these camps. ANC attendance and basic education were associated with SBA. Given the cross‐sectional design, the sampling of selected camps, and imprecise subgroup estimates, these associations should not be interpreted as causal or generalized to all displaced populations in Somalia.

## 1 Introduction

In 2023, an estimated 260,000 women died from causes related to pregnancy and childbirth, and most of these deaths were preventable [1]. Progress has slowed, and the current rate of decline is too slow to reach the Sustainable Development Goal (SDG) target of fewer than 70 maternal deaths per 100,000 live births by 2030 [1]. The proportion of births attended by skilled health personnel is SDG indicator 3.1.2 and a core strategy for preventing maternal deaths [2]. Countries with higher skilled birth attendance (SBA) coverage tend to have lower maternal mortality ratios [3].

The burden is greatest in fragile and conflict‐affected settings. In 2023, 37 countries classified as conflict‐affected or fragile accounted for 64% of global maternal deaths [1]. Conflict and displacement weaken health systems, damage infrastructure, and reduce the availability of skilled health workers [4]. Internally displaced persons (IDPs) often lose income, assets, and social support, which adds to the barriers they face in using maternal and child health services [5].

Somalia has one of the highest maternal mortality ratios in the world, estimated at 692 deaths per 100,000 live births [6]. Nationally, only 20.8% of women deliver in a health facility, and facility delivery is associated with household wealth, antenatal care (ANC) attendance, and perceived distance to services [7]. About 3.5 million people are displaced in Somalia, and around 1.9 million IDPs live in urban centres, including the Daynile and Kahda districts of Banadir region [8]. In IDP camps in Kahda and Deynile, only 25% of women delivered their most recent child in a health facility [6]. Cost, distance, and transport are frequently reported barriers to maternal care among IDPs in Banadir [5].

Across low‐ and middle‐income countries, SBA has been associated with maternal education, ANC attendance, household wealth, parity, and physical access to services [9, 10, 11]. In Somalia, research on maternal health equity has focused mainly on national, regional, and wealth‐related differences [12]. Studies in IDP camps have generally measured place of delivery rather than the type of birth attendant [6]. These indicators are not interchangeable, because the SBA indicator depends on the competence of the attendant rather than on where the birth occurs [2]. Evidence on SBA among displaced women in Mogadishu therefore remains limited.

This study estimated the prevalence of SBA and identified factors associated with SBA among women living in IDP camps in Deynile, Kahda, and Garasbaaley districts of Mogadishu, Somalia.

## 2 Methods

### 2.1 Study Design and Reporting

We conducted a community‐based cross‐sectional study, reported according to the STROBE statement [13]. Statistical reporting follows the SAMPL guidelines and the recommendations of Assel et al. [14, 15].

### 2.2 Study Setting and Period

The study was conducted in IDP camps in three districts of Mogadishu, Banadir region, Somalia: Deynile, Kahda, and Garasbaaley. These districts host some of the largest concentrations of displaced people in the region, most displaced by conflict, recurrent drought, and flooding [8]. Mogadishu hosted more than 2,000 registered IDP sites at the end of 2024, most of them in Daynile and Kahda districts [16]. The camps are densely populated and have limited water, sanitation, and health infrastructure, including maternal health services. Data were collected in February 2026.

### 2.3 Study Population and Eligibility

Women were eligible if they were aged 15–49 years, were living in a selected camp during the study period, had given birth at least once, and provided informed consent. Women who were severely ill or unable to be interviewed because of physical or mental incapacity were excluded. All delivery‐related questions referred to the woman's most recent birth; no restriction was placed on the time elapsed since that birth.

### 2.4 Sample Size

The sample size was calculated with the single population proportion formula, *n* = *Z* ^2^ *p*(1 − *p*)/*d* ^2^, with *Z* = 1.96, *p* = 0.50 (no reliable local estimate was available), and *d* = 0.05, giving 384 [17]. No design effect or non‐response allowance was applied.

### 2.5 Sampling Procedure

A three‐stage sampling procedure was used. In the first stage, Deynile, Kahda, and Garasbaaley districts were selected purposively because they host the largest IDP populations in Mogadishu and are of high humanitarian relevance. In the second stage, IDP camps within each district were identified in collaboration with district authorities, camp management committees, and humanitarian partners, and camps were selected in proportion to their population size and accessibility. In the third stage, the total sample of 384 was allocated to the selected camps in proportion to their estimated number of households (n_i = 384 × N_i/N, where N_i is the number of households in camp i and N is the total across selected camps). Households were then selected by systematic random sampling, with a camp‐specific sampling interval (k = N_i/n_i) and a random start. One eligible woman was interviewed per household; where more than one eligible woman was present, one was selected by lottery.

### 2.6 Data Collection

Data were collected with a structured, interviewer‐administered questionnaire developed from the literature on maternal health service use in humanitarian settings. It covered socio‐demographic characteristics, reproductive history, knowledge of obstetric danger signs, ANC use, delivery practices, and access to services. The questionnaire was prepared in English, translated into Somali, and back‐translated. Trained female data collectors fluent in Somali conducted face‐to‐face interviews in private and recorded responses in KoboToolbox on tablets. Responses to the open question on danger signs were first recorded on paper and then entered into KoboToolbox.

### 2.7 Variables and Definitions

#### 2.7.1 Outcome Variable

Skilled birth attendance (SBA) at the most recent birth was coded 1 if the delivery was assisted by a doctor, nurse, or midwife, and 0 if it was assisted by a traditional birth attendant (TBA) or a family member [2]. Where more than one attendant was recorded, the most qualified attendant was used, following the Demographic and Health Surveys (DHS) convention [18]. SBA refers to the type of attendant and is reported separately from place of delivery (“facility delivery”).

#### 2.7.2 Independent Variables

Candidate variables were maternal age, education, marital status, occupation, parity, age at first marriage, age at first birth, any ANC, income regularity, receipt of food or cash assistance, travel time to the nearest facility, and usual mode of transport. Number of ANC visits and ANC provider were described but not modelled because they apply only to ANC attenders.

#### 2.7.3 Categorization of Continuous Variables

Maternal age was grouped in 5‐year bands as in DHS reports, with 40–49 years combined because of small numbers [18]. Age at first marriage was grouped as < 15, 15–17, and ≥ 18 years, reflecting the definition of child marriage as marriage before 18 years [19]. Age at first birth was grouped as < 18, 18–19, and ≥ 20 years to separate adolescent from adult first births. Parity was grouped as 1–2, 3–4, and ≥ 5 births (grand multiparity). Travel time was recorded in the questionnaire as ≤ 30 or > 30 min. Medians and interquartile ranges (IQRs) are also reported for the continuous variables because their distributions were skewed.

### 2.8 Data Quality Assurance and Cleaning

The questionnaire was pre‐tested among 5% of the sample in a comparable IDP setting not included in the study. Data collectors and supervisors received training on the study procedures, the questionnaire, interviewing technique, and research ethics. KoboToolbox forms included skip logic and mandatory fields, and supervisors reviewed submissions daily.

Before analysis, the exported dataset (384 records) was cleaned using prespecified rules, which are documented in Supplementary Table S2. Two women whose recorded ages (60 and 75 years) were outside the eligibility range were excluded. A non‐numeric age entry was set to missing. Biologically implausible values (age at marriage or first birth < 12 years, parity > 12, or age at first birth greater than current age; 6 values in 4 records) were set to missing. Where more than one option had been recorded for a single‐choice question (35 responses across 9 variables), the first recorded option was used, except for birth attendant, where the most qualified attendant was used.

### 2.9 Statistical Analysis

Analyses were performed in Python version 3.12 using the statsmodels package version 0.14 [20]. Categorical variables are reported as *n*/*N* (%), with Wilson score 95% CIs for key indicators [21]; skewed continuous variables are reported as medians and IQRs.

#### 2.9.1 Prespecified Analysis

The primary analysis was a multivariable binary logistic regression model of SBA. Each candidate variable was first examined in bivariable logistic regression, and variables with an overall likelihood‐ratio *p* < 0.20 were entered into the multivariable model [22, 23]. Results are presented as crude odds ratios (CORs) and adjusted odds ratios (AORs) with 95% CIs, and *P* values from Wald tests. All tests were two‐sided, with *α* = 0.05 set a priori.

#### 2.9.2 Model Diagnostics

Overall model significance was assessed with the likelihood‐ratio *χ* ^2^ test, calibration with the Hosmer–Lemeshow test (10 groups), and discrimination with the area under the receiver operating characteristic curve (AUC), whose 95% CI was obtained from 2000 bootstrap resamples [23]. We also report classification accuracy, sensitivity, and specificity at a cut‐off of 0.5, and McFadden and Nagelkerke pseudo *R* ^2^. Multicollinearity was assessed with variance inflation factors (VIFs), with VIF > 5 considered notable. Influential observations were identified by standardized Pearson residuals (|r | > 3) and Pregibon's delta‐beta (> 1), and the model was refitted without them.

#### 2.9.3 Events Per Variable and Sensitivity Analyses

Because the number of events per estimated parameter (EPV) was below 10 [24, 25]. the model was refitted with Firth's penalized‐likelihood logistic regression, which reduces small‐sample bias [26]. A second sensitivity analysis treated the 12 missing ANC responses as “no ANC”.

#### 2.9.4 Missing Data

The primary model was a complete‐case analysis. Missing values are reported for each variable in the tables.

#### 2.9.5 Exploratory Analyses

Two post hoc crude analyses examined the stability of estimates for sparse categories: age collapsed into three groups (15–24, 25–34, and 35–49 years), and education dichotomized as any versus none. Fisher's exact test was used for comparisons with small cell counts. No interaction terms were tested, given the small number of events.

#### 2.9.6 Survey Design

Analyses were unweighted and did not account for clustering of women within camps.

### 2.10 Ethical Considerations

Ethical approval was obtained from the Somali National University Institutional Review Board (Ref: JUS/KCC&CT/XT/0058/2026). Permission to conduct the study was also obtained from district authorities and camp management committees. All participants were informed about the purpose of the study, assured of confidentiality, and provided written informed consent before participation. Participation was voluntary, and respondents were informed of their right to withdraw at any time without consequences. Interviews were conducted in private by female data collectors, and no personal identifiers were retained in the analysis dataset. The study was conducted in accordance with the Declaration of Helsinki.

## 3 Results

### 3.1 Participants

A total of 384 women were interviewed. Of these, 2 were excluded because their ages were outside the eligibility range, leaving 382 for analysis. The birth attendant was missing for 1 woman, so SBA analyses included 381 women. The multivariable model included 366 women with complete data on all model variables.

The sociodemographic and reproductive characteristics of the participants are presented in Table 1. The median age was 28 years (IQR 25–35; range 17–48). Most women were married (298/382, 78.0%) and illiterate (281/382, 73.6%); only 6 (1.6%) had primary education. The median age at first marriage was 16 years (IQR 15–17): 292 of 380 women (76.8%) married before 18 years, and 176 of 379 (46.4%) had their first birth before 18 years. The median parity was 5 (IQR 3–6). Household income was irregular for 340 women (89.0%), and 119 (31.2%) received food or cash assistance at least occasionally.

**Table 1:** Characteristics of internally displaced women in IDP camps in Deynile, Kahda, and Garasbaaley districts, Mogadishu, February 2026 (*N* = 382).

Characteristic	Category	*n* (%)
Age (years) (*n* = 381)^a^	15–19	20 (5.2)
	20–24	63 (16.5)
	25–29	130 (34.1)
	30–34	66 (17.3)
	35–39	84 (22.0)
	40–49	18 (4.7)
	Median (IQR)	28 (25–35)
Marital status	Married	298 (78.0)
	Divorced	72 (18.8)
	Widowed	12 (3.1)
Education	Illiterate	281 (73.6)
	Quranic education	95 (24.9)
	Primary (grades 1–8)	6 (1.6)
Occupation (*n* = 381)^a^	Housewife/no paid work	235 (61.7)
	Washing clothes for others	62 (16.3)
	Daily wage labour	35 (9.2)
	Small business	21 (5.5)
	Street vending	16 (4.2)
	Skilled work	9 (2.4)
	Other	3 (0.8)
Husband's occupation (*n* = 297)^b^	Daily wage labour	120 (40.4)
	Skilled work	85 (28.6)
	Small business	31 (10.4)
	Driver/transport	25 (8.4)
	Street vending	21 (7.1)
	Unemployed	7 (2.4)
	Other	8 (2.7)
Age at first marriage (*n* = 380)^a^	< 15 years	62 (16.3)
	15–17 years	230 (60.5)
	≥ 18 years	88 (23.2)
	Median (IQR)	16 (15–17)
Age at first birth (*n* = 379)^a^	< 18 years	176 (46.4)
	18–19 years	123 (32.5)
	≥ 20 years	80 (21.1)
	Median (IQR)	18 (16–19)
Parity (*n* = 380)^a^	1–2	70 (18.4)
	3–4	110 (28.9)
	≥ 5	200 (52.6)
	Median (IQR)	5 (3–6)
Main household income source	Daily labour	312 (81.7)
	No regular income	38 (9.9)
	Small business	29 (7.6)
	Private sector employment	3 (0.8)
Income regularity	Irregular	340 (89.0)
	Regular	42 (11.0)
Food/cash assistance	No	263 (68.8)
	Yes, sometimes	108 (28.3)
	Yes, regularly	11 (2.9)

Values are *n* (%) with *N* = 382 as denominator unless stated; IQR, interquartile range. Percentages may not total 100 because of rounding.

^a^ Denominator excludes missing or implausible values set to missing during cleaning (Supplementary Table S2).

^b^ Reported by married women only.

### 3.2 Knowledge of Obstetric Danger Signs

Of 381 women, 85 (22.3%; 95% CI: 18.4–26.8) reported knowing at least one danger sign of pregnancy or delivery, and 83 named specific signs (Table 2). The most frequently named signs were severe headache (77/83, 92.8%) and high fever (74/83, 89.2%). Forty‐five of the 83 women (54.2%) named all ten signs listed on the data collection form, which may indicate prompting during the interview.

**Table 2:** Knowledge of obstetric danger signs among internally displaced women, Mogadishu, February 2026.

Indicator	Category	*n* (%)
Aware of ≥ 1 danger sign (*n* = 381)	Yes	85 (22.3)
	No	296 (77.7)
Danger signs named (*n* = 83)^a^	Severe headache	77 (92.8)
	High fever	74 (89.2)
	Premature labour	57 (68.7)
	Maternal injury	55 (66.3)
	Prolonged labour	52 (62.7)
	Breech presentation	52 (62.7)
	Cord prolapse	52 (62.7)
	Postpartum haemorrhage	52 (62.7)
	Convulsions	52 (62.7)
	Foul‐smelling discharge	50 (60.2)
	Absent foetal movement	2 (2.4)

^a^ Among women aware of at least one sign who named specific signs (2 aware women did not name any). Multiple responses allowed; percentages do not total 100. Forty‐five women named all ten listed signs.

### 3.3 Maternal Health Service Use and Prevalence of SBA

Only 42 of 370 women (11.4%; 95% CI: 8.5–15.0) received any ANC during their most recent pregnancy (Table 3). Among ANC attenders, the number of visits was recorded for 20 women, of whom 9 (45.0%) had four or more visits; the ANC provider was recorded for 27 women, most often an assistant nurse‐midwife (15/27, 55.6%).

**Table 3:** Antenatal care, delivery, and access characteristics among internally displaced women, Mogadishu, February 2026.

Indicator	Category	*n* (%)
Any ANC, most recent pregnancy (*n* = 370)^a^	Yes	42 (11.4)
	No	328 (88.6)
Number of ANC visits (*n* = 20)^b^	1	1 (5.0)
	2	8 (40.0)
	3	2 (10.0)
	≥ 4	9 (45.0)
ANC provider (*n* = 27)^b^	Assistant nurse‐midwife	15 (55.6)
	Maternal and child health worker	10 (37.0)
	Other	2 (7.4)
Place of most recent delivery (*n* = 382)	Home	255 (66.8)
	Health facility	76 (19.9)
	TBA's place	51 (13.4)
Birth attendant (*n* = 381)^a^	Skilled birth attendant^c^	68 (17.8)
	Traditional birth attendant	295 (77.4)
	Family member	18 (4.7)
Reason for using SBA (*n* = 65)^d^	Safe delivery	65 (100)
Reasons for not using SBA (*n* = 308)^e	Unable to pay delivery costs	157 (51.0)
	Inadequate transportation	97 (31.5)
	Distance to health facility	28 (9.1)
	Other	61 (19.8)
Travel time to nearest facility (*n* = 382)	≤ 30 min	203 (53.1)
	> 30 min	179 (46.9)
Usual mode of transport (*n* = 382)	Walking	190 (49.7)
	Private vehicle (bajaaj)	161 (42.1)
	Public transport	31 (8.1)

Abbreviations: ANC, antenatal care; SBA, skilled birth attendance; TBA, traditional birth attendant.

^a^ Excludes missing responses (ANC 12; attendant 1).

^b^ Among the 42 ANC attenders with a recorded response (visits missing for 22; provider for 15).

^c^ Doctor, nurse, or midwife.

^d^ Among 68 SBA‐attended women; 3 missing. ^e Among 313 women without SBA; 5 missing; multiple responses allowed (31 women gave more than one), so percentages do not total 100.

#### 3.3.1 The Prevalence of SBA Was 17.8% (68/381; 95% CI: 14.3–22.0)

Traditional birth attendants assisted 295 deliveries (77.4%; 95% CI: 73.0–81.3) and family members 18 (4.7%). Most women delivered at home (255/382, 66.8%; 95% CI: 61.9–71.3); 76 (19.9%; 95% CI: 16.2–24.2) delivered in a health facility and 51 (13.4%) at a TBA's place (Figure 1). The two indicators did not fully overlap: 64 of the 68 SBA‐attended births occurred in a facility, while 11 of the 76 facility births were attended by a TBA. Among 308 women who gave a reason for not using SBA, the most common reasons were inability to pay (157, 51.0%) and inadequate transport (97, 31.5%).

**Figure 1:**
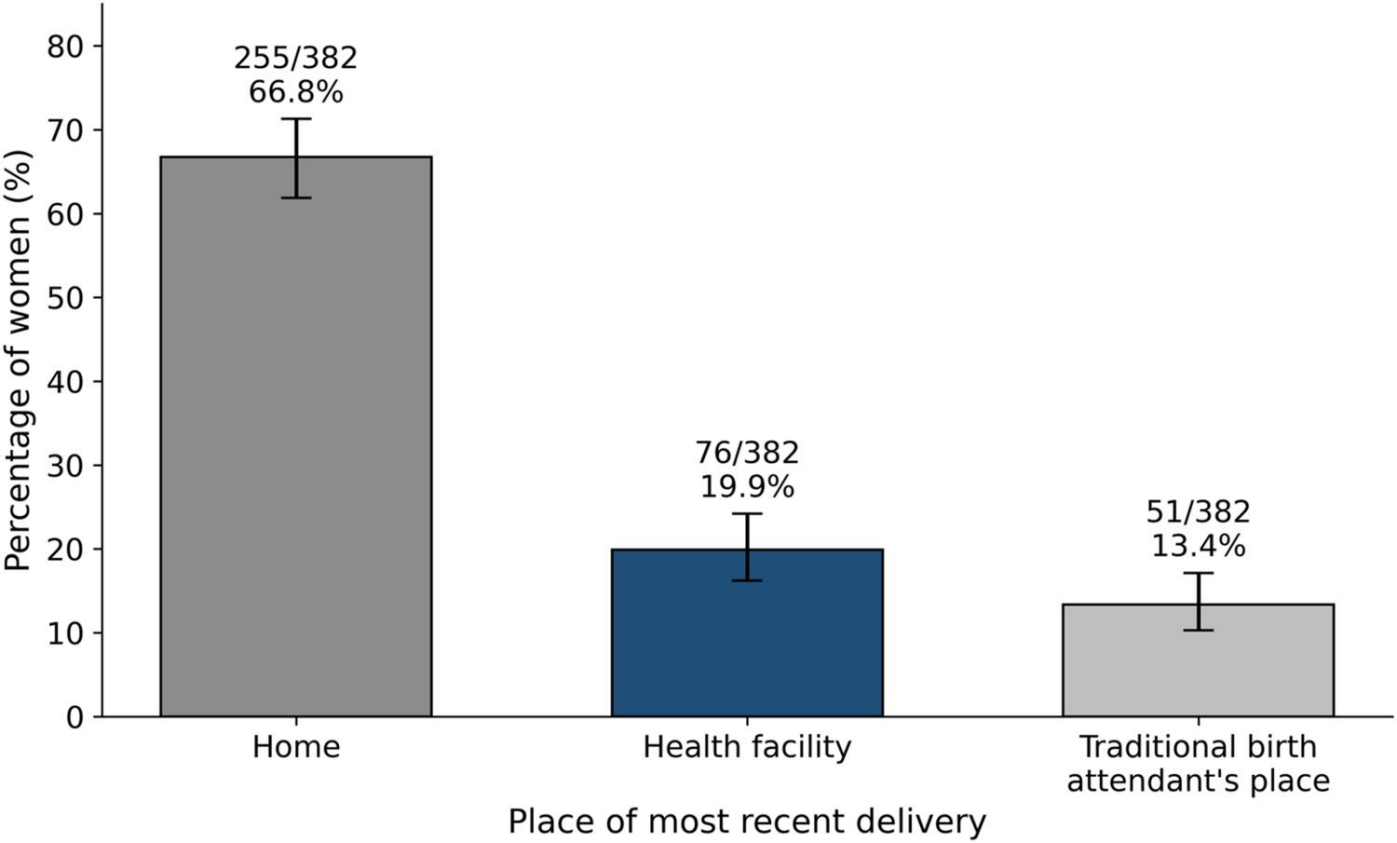
Place of most recent delivery among internally displaced women in IDP camps in Deynile, Kahda, and Garasbaaley districts, Mogadishu, February 2026 (N = 382). Bars show percentages; labels give *n*/*N* and percentages; error bars are 95% Wilson CIs. Place of delivery differs from birth attendant: 76 women delivered in a facility, whereas 68 were assisted by a skilled birth attendant.

### 3.4 Factors Associated With SBA

Seven variables met the screening criterion (*p* < 0.20): maternal age, education, age at first marriage, age at first birth, ANC, travel time, and mode of transport. Marital status, occupation, parity, income regularity, and food or cash assistance did not (all *p* ≥ 0.31; Supplementary Table S1). The multivariable model included 366 women and 67 events (Table 4, Figure 2).

**Table 4:** Crude and adjusted associations with skilled birth attendance among internally displaced women, Mogadishu, February 2026 (crude: *n* = 370–381; adjusted: *n* = 366, 67 events).

Variable	Category	SBA, *n*/*N* (%)	COR (95% CI)	*p*	AOR (95% CI)	*p*
Age (years)	15–19 (ref)	9/20 (45.0)	1.00	—	1.00	—
	20–24	11/63 (17.5)	**0.26 (0.09–0.77)**	**0.02**	0.33 (0.09–1.23)	0.10
	25–29	23/130 (17.7)	**0.26 (0.10–0.71)**	**0.008**	0.42 (0.12–1.39)	0.15
	30–34	7/66 (10.6)	**0.15 (0.04–0.47)**	**0.001**	**0.22 (0.06–0.87)**	**0.03**
	35–39	16/83 (19.3)	**0.29 (0.10–0.82)**	**0.02**	0.43 (0.12–1.50)	0.19
	40–49	2/18 (11.1)	**0.15 (0.03–0.85)**	**0.03**	0.15 (0.02–1.03)	0.05
Education	Illiterate (ref)	34/281 (12.1)	1.00	—	1.00	—
	Quranic	31/94 (33.0)	**3.57 (2.04–6.26)**	**< 0.001**	**3.13 (1.61–6.10)**	**< 0.001**
	Primary^a^	3/6 (50.0)	**7.26 (1.41–37.45)**	**0.02**	**6.13 (1.05–35.91)**	**0.04**
Age at first marriage	< 15 years (ref)	17/61 (27.9)	1.00	—	1.00	—
	15–17 years	37/230 (16.1)	**0.50 (0.26–0.96)**	**0.04**	0.67 (0.29–1.53)	0.34
	≥ 18 years	14/88 (15.9)	0.49 (0.22–1.09)	0.08	1.45 (0.25–8.50)	0.68
Age at first birth	< 18 years (ref)	39/175 (22.3)	1.00	—	1.00	—
	18–19 years	17/123 (13.8)	0.56 (0.30–1.04)	0.07	0.56 (0.25–1.26)	0.16
	≥ 20 years	12/80 (15.0)	0.62 (0.30–1.25)	0.18	0.29 (0.05–1.76)	0.18
Any ANC	No (ref)	49/328 (14.9)	1.00	—	1.00	—
	Yes	18/42 (42.9)	**4.27 (2.16–8.45)**	**< 0.001**	**3.17 (1.27–7.94)**	**0.01**
Travel time to facility	> 30 min (ref)	41/179 (22.9)	1.00	—	1.00	—
	≤ 30 min	27/202 (13.4)	**0.52 (0.30–0.89)**	**0.02**	0.59 (0.23–1.49)	0.26
Mode of transport	Private vehicle (ref)	28/161 (17.4)	1.00	—	1.00	—
	Public transport^a^	2/31 (6.5)	0.33 (0.07–1.45)	0.14	**0.14 (0.02–0.77)**	**0.02**
	Walking	38/189 (20.1)	1.20 (0.70–2.05)	0.52	0.69 (0.27–1.77)	0.44

Abbreviations: ANC, antenatal care; AOR, adjusted odds ratio; AUC, area under the ROC curve; CI, confidence interval; COR, crude odds ratio; EPV, events per variable; ref, reference; SBA, skilled birth attendance; VIF, variance inflation factor.

Model: binary logistic regression including all variables shown; complete‐case *n* = 366; events = 67; parameters = 15; EPV = 4.5. Likelihood‐ratio *χ* ^2^ = 54.9, *p* < 0.001; Hosmer–Lemeshow *χ* ^2^ = 15.97, df = 8, *p* = 0.04; AUC = 0.79 (95% CI 0.73–0.84); Nagelkerke pseudo *R* ^2^ = 0.23; correctly classified = 81.4%; mean VIF = 3.20 (maximum 7.65).

Crude estimates use all women with non‐missing data for that variable. *P* values from Wald tests (two‐sided). Bold indicates *p* < 0.05 (95% CI excludes 1), applied identically in both columns.

^a^ Sparse category (6 and 31 women, with 3 and 2 events, respectively); estimates are imprecise. The adjusted *p* = 0.05 for age 40–49 years was 0.054 before rounding.

**Figure 2:**
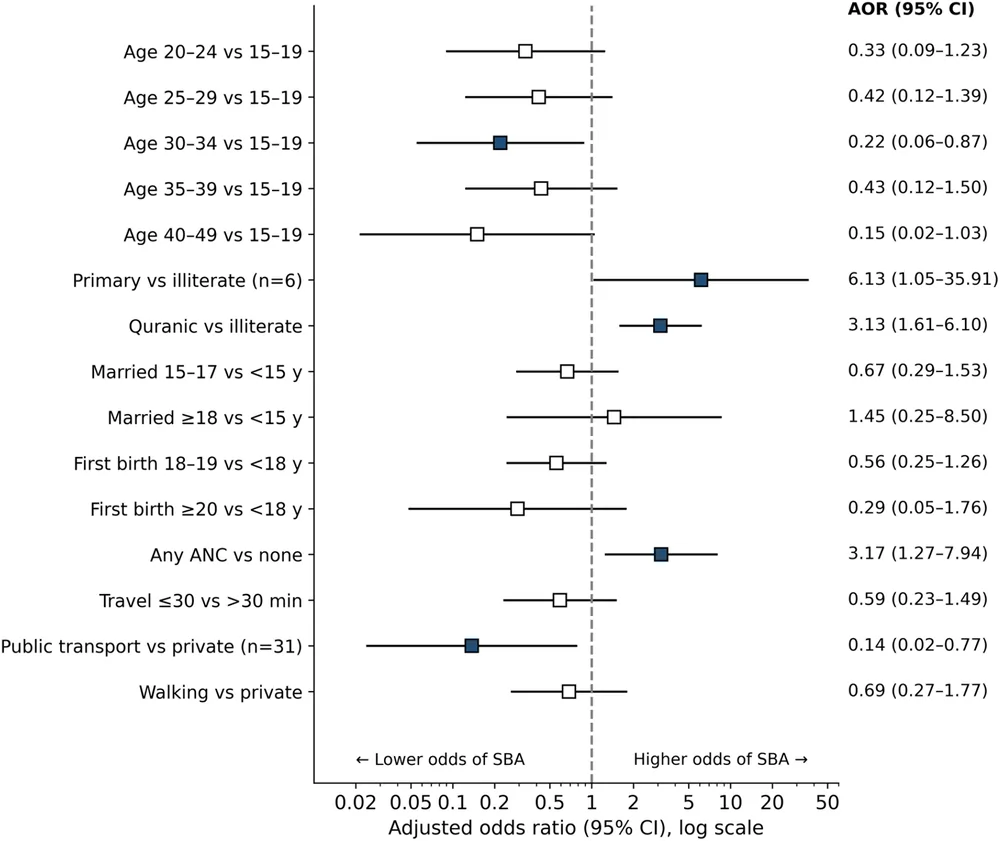
Adjusted odds ratios for skilled birth attendance among internally displaced women, Mogadishu, February 2026 (*n* = 366; 67 events). Squares show adjusted odds ratios from the multivariable logistic regression model in Table 4, and horizontal lines show 95% CIs on a logarithmic scale. Filled squares indicate CIs that exclude 1 (dashed line = no association); values to the right indicate higher odds of SBA than the reference category. Wide intervals for primary education and public transport reflect very small subgroups.

#### 3.4.1 Antenatal Care

SBA was reported by 18 of 42 women who attended ANC (42.9%) compared with 49 of 328 who did not (14.9%) (AOR 3.17; 95% CI: 1.27–7.94; *p* = 0.01).

#### 3.4.2 Education

SBA was reported by 31 of 94 women with Quranic education (33.0%) compared with 34 of 281 illiterate women (12.1%) (AOR 3.13; 95% CI: 1.61–6.10; *p* < 0.001). Primary education was also associated with SBA (AOR 6.13; 95% CI: 1.05–35.91; *p* = 0.04), but this estimate rests on 3 events among 6 women and was not statistically significant in the Firth‐penalized model. In an exploratory analysis, 34 of 100 women with any education (34.0%) had SBA, compared with 34 of 281 (12.1%) without (COR 3.74; 95% CI: 2.16–6.47).

#### 3.4.3 Transport

Women who usually travelled by public transport had lower adjusted odds of SBA than those using a private vehicle (AOR 0.14; 95% CI: 0.02–0.77; *p* = 0.02). Only 2 of these 31 women had SBA (6.5%), and the crude association was not significant (Fisher's exact *p* = 0.18), so this estimate is imprecise.

#### 3.4.4 Maternal Age

All older age groups had lower crude odds of SBA than women aged 15–19 years, a small reference group with a high proportion of SBA (9/20, 45.0%). After adjustment, only women aged 30–34 years differed significantly (AOR 0.22; 95% CI: 0.06–0.87; *p* = 0.03), while adjacent groups had similar point estimates (0.15–0.43) with CIs including 1, and the variable as a whole was not significant (likelihood‐ratio *p* = 0.23). In an exploratory crude analysis with broader groups, SBA did not differ significantly between women aged 25–34 years (30/196, 15.3%; COR 0.57; 95% CI: 0.30–1.08) or 35–49 years (18/101, 17.8%; COR 0.68; 95% CI: 0.33–1.40) and those aged 15–24 years (20/83, 24.1%). The estimate for 30–34 years is therefore more consistent with small‐sample instability than with a distinct age effect.

#### 3.4.5 Other Factors

Women living within 30 min of a facility had lower crude odds of SBA (27/202 [13.4%] vs. 41/179 [22.9%]; COR 0.52; 95% CI: 0.30–0.89), but this association was attenuated after adjustment (AOR 0.59; 95% CI: 0.23–1.49). Age at first marriage and age at first birth were not associated with SBA after adjustment.

#### 3.4.6 Model Performance

The model was statistically significant (likelihood‐ratio *χ* ^2^ = 54.9, df = 15, *p* < 0.001). Discrimination was good (AUC 0.79; 95% CI: 0.73–0.84), but the Hosmer–Lemeshow test suggested some lack of calibration (*χ* ^2^ = 15.97, df = 8, *p* = 0.04). Nagelkerke pseudo *R* ^2^ was 0.23 and McFadden pseudo *R* ^2^ was 0.16. At a cut‐off of 0.5, the model correctly classified 81.4% of women, with sensitivity 16.4% and specificity 96.0%. The mean VIF was 3.20; the highest VIFs were for age at first marriage ≥ 18 years (7.65) and age at first birth ≥ 20 years (6.76), reflecting the expected correlation between these variables, which may explain their wide CIs. Six observations had standardized Pearson residuals > 3, but none had a delta‐beta > 1 (maximum 0.78); refitting without them strengthened the main associations without changing their direction. With 67 events and 15 parameters, the EPV was 4.5.

#### 3.4.7 Sensitivity Analyses

In the Firth‐penalized model, the associations for ANC (AOR 2.99 (1.22–7.34); *p* = 0.02), Quranic education (2.95 (1.54–5.65); *p* = 0.001), and public transport (0.18 (0.04–0.89); *p* = 0.04) were similar to the main model, whereas the CI for primary education included 1 (5.50 (0.95–31.85); *p* = 0.06). Treating missing ANC responses as “no ANC” (*n* = 376; 68 events) gave consistent results (ANC: AOR 3.33 (1.37–8.11); *p* = 0.008; Quranic education: 2.81 (1.47–5.37); *p* = 0.002).

## 4 Discussion

### 4.1 Summary of Main Findings

In this study, fewer than one in five women living in IDP camps in Deynile, Kahda, and Garasbaaley districts gave birth with a skilled attendant (17.8%). Traditional birth attendants (TBAs) attended more than three‐quarters of births, and only 11.4% of women received any ANC. After adjustment, ANC attendance and Quranic education were associated with higher odds of SBA. Estimates for primary education, public transport, and individual age groups were based on small numbers and should be interpreted with caution.

### 4.2 Prevalence of SBA

The prevalence of SBA in this study was lower than the national facility delivery rate of 20.8% [7]. and the 24.2% reported in an equity analysis of the same survey [27]. It was also lower than the 25% facility delivery reported in IDP camps in Kahda and Deynile in 2023 [6]. These comparisons should be made cautiously, because the earlier studies measured place of delivery rather than attendant type and used different sampling methods and recall periods. Our data show why this distinction matters: 11 of 76 facility births were attended by a TBA, and 4 skilled births took place at home. The SBA indicator is intended to capture care by competent personnel, not simply the place of birth [2]. The reasons for TBA‐attended facility births could not be determined from our data and deserve further study.

### 4.3 Antenatal Care

Women who attended ANC were more likely to give birth with a skilled attendant than those who did not (42.9% vs. 14.9%; AOR 3.17). A similar association between ANC and facility delivery was reported in the 2020 Somali Health and Demographic Survey [7], and ANC attendance was linked to later use of maternal services in a conflict‐affected setting [28]. ANC visits may offer birth preparedness counselling and information about where to give birth [29]. However, studies in northern Uganda and rural Tanzania show that high ANC use does not always lead to SBA [29, 30]. ANC consultations are also often too short to cover birth planning in depth [31].

An alternative explanation is self‐selection: women who are already able or inclined to use formal services may seek both ANC and SBA. Our cross‐sectional design cannot distinguish between these explanations. ANC coverage itself was very low. A systematic review of 37 fragile and conflict‐affected settings identified cost, distance, insecurity, and service quality as barriers to ANC [32]. Whether expanding ANC in the camps would increase SBA should be tested in intervention studies.

### 4.4 Maternal Education

Women with Quranic education were more likely to have SBA than illiterate women (33.0% vs. 12.1%; AOR 3.13), and this association remained in all sensitivity analyses. Education has been associated with facility delivery in Somalia and Somaliland [27, 33] and with SBA in other settings [9, 10]. Quranic education is rarely reported as a separate category in maternal health studies. Possible explanations include basic literacy, greater confidence in dealing with services, and wider social networks, but these were not measured. Quranic education may also be a marker of higher household socioeconomic status, which we could not fully adjust for. The estimate for primary education was based on 3 events among 6 women, was imprecise, and was not significant in the Firth model, so no conclusion can be drawn from it.

### 4.5 Cost and Access

Inability to pay was the most frequently reported reason for not using a skilled attendant (51.0%), followed by inadequate transport (31.5%). Similar financial and transport barriers have been reported among IDPs in Banadir region [5, 6]. Although 89.0% of households had irregular income, income regularity was not associated with SBA in our analysis. This single measure may not adequately capture household economic status, and no wealth index was available.

The association with public transport (AOR 0.14) was based on only 2 women with SBA among 31, and the crude association was not significant. It should therefore be regarded as uncertain. Women living within 30 min of a facility had lower crude odds of SBA, but this association was attenuated after adjustment. This unexpected finding may reflect confounding or the location of camps relative to facilities, which we could not examine. Distance has been associated with lower facility delivery in Somalia [7] and with dropout from the maternal continuum of care in Ethiopia [34].

### 4.6 Knowledge of Danger Signs

Only 22.3% of women reported knowing at least one danger sign of pregnancy or delivery. Among women who named signs, recognition appeared broad, but 45 of 83 named all ten signs listed on the form. This pattern suggests that some responses were prompted, so knowledge in this group may be overestimated. Evidence from other settings suggests that knowledge alone may not translate into SBA where cost and transport barriers remain [30].

### 4.7 Factors Not Associated With SBA

Parity, marital status, occupation, food or cash assistance, income regularity, and age at first marriage or first birth were not associated with SBA. In the national survey, higher parity and early marriage were associated with lower facility delivery [7]. The difference may reflect the relative homogeneity of this displaced population or the limited sample size. For maternal age, only the 30–34‐year group differed significantly from the reference group, and age was not significant overall. The reference group was small (20 women), with a high proportion of SBA (45.0%), so this finding most likely reflects statistical instability rather than a true age effect.

### 4.8 Implications

These findings suggest priorities for programme evaluation rather than proven interventions. Extending ANC services into the camps through outreach or mobile teams could increase contact with skilled providers [32]. Because cost was the most commonly reported barrier, approaches that reduce out‐of‐pocket costs, such as free delivery services or vouchers, merit evaluation in these camps [5]. Improving referral transport may also be important, although our transport estimates were imprecise.

TBAs attended most births in this population. In a refugee camp in Kenya, TBAs have supported mothers during pregnancy and after birth [35]. Our study did not assess the role of TBAs, so any formal role for them in Mogadishu IDP camps should be evaluated for safety and effectiveness before it is adopted.

### 4.9 Strengths and Limitations

This study has several strengths. It focused specifically on displaced women, measured attendant type rather than place of delivery, used electronic data collection with documented data cleaning, and included model diagnostics and sensitivity analyses.

The study also has important limitations. First, the cross‐sectional design does not allow causal inference or establish temporal order. Second, no limit was placed on the time since the most recent birth, so recall bias is possible. Third, social desirability bias may have affected reports of ANC and reasons for non‐use of SBA, and responses on danger signs may have been prompted. Fourth, only women available at home during data collection were interviewed, and districts and camps were partly selected for accessibility, which may introduce selection bias. Fifth, residual confounding is likely, because household wealth, husband's education, women's decision‐making autonomy, cultural beliefs, and facility quality were not measured. Sixth, the model had only 4.5 events per parameter, several subgroups were very small, and the Hosmer–Lemeshow test suggested some miscalibration. Seventh, ANC visit and provider data were incomplete. Eighth, the analysis did not account for clustering within camps, so confidence intervals may be too narrow. Finally, the study included selected camps in three districts of Mogadishu, so the findings should not be generalized to all displaced populations in Somalia, particularly those in rural settlements.

## 5 Conclusion

Skilled birth attendance was very low among internally displaced women in IDP camps in Deynile, Kahda, and Garasbaaley districts of Mogadishu, and most births were attended by traditional birth attendants. ANC attendance and Quranic education were associated with higher odds of SBA, while inability to pay was the most commonly reported barrier. Because of the cross‐sectional design and small subgroups, these associations should be interpreted cautiously. Longitudinal and intervention studies with larger samples are needed to test whether expanding ANC and reducing delivery costs increase SBA in displacement settings.

## Author Contributions

**Maryama Ahmed Ali:** conceptualization; methodology; investigation; project administration; writing – original draft. **Yusuf Hared Abdi:** formal analysis; software; data curation; visualization. **Abdisalan Abdikarin Mohamed:** investigation; data curation; validation. **Yaxye Abuukar Hassan:** investigation; data curation; validation. **Abdiqani Mohamed Jama:** investigation; data curation; validation. **Sadio Abdulahi Osman:** investigation; data curation; visualization. **Sharmake Gaiye Bashir:** formal analysis; software; visualization; data curation. **Yakub Burhan Abdullahi:** formal analysis; software; visualization; data curation. **Naima Ibrahim Ahmed:** writing – review and editing; validation. **Ilyas Abdullahi Khalif:** validation; writing – review and editing. **Mohamed Sharif AbdI:** validation; writing – review and editing. **Ahmed Abdinasir Abdulle:** methodology; supervision; writing – review and editing. **Nuradin Abdullahi Sheikh Rashid:** methodology; supervision; writing – review and editing. All authors reviewed and approved the final version of the manuscript and agree to be accountable for all aspects of the work.

## Funding

The authors have nothing to report.

## Conflicts of Interest

The authors declare no conflicts of interest.

## Transparency Statement

The lead author and manuscript guarantor affirm that this manuscript is an honest, accurate, and transparent account of the study being reported; that no important aspects of the study have been omitted; and that any discrepancies from the study as originally planned have been explained.

## Supporting information


Supporting File

## Data Availability

The de‐identified data supporting the findings of this study are available from the corresponding author upon reasonable request and in line with the Somali National University Research Ethics Committee approval (Ref: JUS/KCC& CT/XT/0058/2026). The data that support the findings of this study are available on request from the corresponding author. The data are not publicly available due to privacy or ethical restrictions.
