# Supplementary material for: Factors Associated With Skilled Birth Attendance Among Internally Displaced Women in Mogadishu, Somalia: A Community‐Based Cross‐Sectional Study
Source: Health Sci Rep. 2026 Oct 13;9(10):e73379. doi: 10.1002/hsr2.73379 (PMC13659272; doi:10.1002/hsr2.73379)
Supplement: Supplementary file 1 [file HSR2-9-e73379-s001.docx]

**Supplementary Table S1.** Bivariable associations with skilled birth attendance for variables not retained in the multivariable model (N = 381).

| **Variable** | **Category** | **SBA, n/N (%)** | **COR (95% CI)** | **P** | **Overall P**^a^ |
| --- | --- | --- | --- | --- | --- |
| Marital status | Married (ref) | 53/298 (17.8) | 1.00 | — | .95 |
|  | Divorced or widowed | 15/83 (18.1) | 1.02 (0.54–1.92) | .95 |  |
| Occupation | No paid work (ref) | 43/235 (18.3) | 1.00 | — | .77 |
|  | Any paid work | 25/146 (17.1) | 0.92 (0.54–1.59) | .77 |  |
| Parity | 1–2 (ref) | 15/70 (21.4) | 1.00 | — | .31 |
|  | 3–4 | 23/110 (20.9) | 0.97 (0.47–2.02) | .93 |  |
|  | ≥5 | 30/199 (15.1) | 0.65 (0.33–1.30) | .22 |  |
| Income regularity | Irregular (ref) | 59/340 (17.4) | 1.00 | — | .48 |
|  | Regular | 9/41 (22.0) | 1.34 (0.61–2.95) | .47 |  |
| Food/cash assistance | No (ref) | 48/263 (18.3) | 1.00 | — | .76 |
|  | Yes | 20/118 (16.9) | 0.91 (0.52–1.62) | .76 |  |

^a^ Likelihood-ratio test for the variable as a whole; variables with P ≥ .20 were not entered into the multivariable model. Parity: n = 379 (implausible values set to missing). Abbreviations as in Table 4.

**Supplementary Table S2.** Data cleaning log (384 exported records).

| **Variable** | **Issue identified** | **Action** |
| --- | --- | --- |
| Age | 2 records with age 60 and 75 years (outside 15–49 inclusion criterion) | Excluded from analysis |
| Age | 1 record with non-numeric entry ("3r") | Set to missing |
| Age at marriage / first birth / parity | 6 implausible values in 4 records (marriage at 2 and 9 years; first birth at 11 years; parity 17 at age 22; parity 18; first birth after current age) | Set to missing |
| Single-choice items | 35 responses with more than one option recorded (marital status 1, education 2, occupation 2, husband's occupation 3, income source 3, assistance 1, transport 19, danger-sign awareness 2, attendant 2) | First recorded option used; attendant classified by most qualified provider (DHS convention) |
| Birth attendant | 1 record with no attendant recorded | Set to missing |
| ANC | 12 eligible records with no response | Set to missing; sensitivity analysis treats as "No" |
| ANC visits / provider | Missing for 22 and 15 of 42 ANC attenders | Reported with actual denominators |
